# Structure-Based Modulation of the Ligand Sensitivity of a Tomato Dimeric Abscisic Acid Receptor Through a Glu to Asp Mutation in the Latch Loop

**DOI:** 10.3389/fpls.2022.884029

**Published:** 2022-06-06

**Authors:** Lourdes Infantes, Maria Rivera-Moreno, Miguel Daniel-Mozo, Juan Luis Benavente, Javier Ocaña-Cuesta, Alberto Coego, Jorge Lozano-Juste, Pedro L. Rodriguez, Armando Albert

**Affiliations:** ^1^Instituto de Química Física Rocasolano, Consejo Superior de Investigaciones Científicas, Madrid, Spain; ^2^Instituto de Biología Molecular y Celular de Plantas, Consejo Superior de Investigaciones Científicas-Universidad Politécnica de Valencia, Valencia, Spain

**Keywords:** abiotic stress, abscisic acid, plant biology, protein crystallography, signal transduction, structural biology

## Abstract

The binding of the plant phytohormone Abscisic acid (ABA) to the family of ABA receptors (PYR/PYL/RCAR) triggers plant responses to abiotic stress. Thus, the implementation of genetic or chemical strategies to modulate PYR/PYL activity might be biotechnologically relevant. We have employed the available structural information on the PYR/PYL receptors to design SlPYL1, a tomato receptor, harboring a single point mutation that displays enhanced ABA dependent and independent activity. Interestingly, crystallographic studies show that this mutation is not directly involved in ABA recognition or in the downstream phosphatase (PP2C) inhibitory interaction, rather, molecular dynamic based ensemble refinement restrained by crystallographic data indicates that it enhances the conformational variability required for receptor activation and it is involved in the stabilization of an active form of the receptor. Moreover, structural studies on this receptor have led to the identification of niacin as an ABA antagonist molecule *in vivo*. We have found that niacin blocks the ABA binding site by mimicking ABA receptor interactions, and the niacin interaction inhibits the biochemical activity of the receptor.

## Introduction

Abiotic stresses derived from global warming are threatening natural resources and food production; of them, drought is central as irrigated lands produce one-third of the world’s food^1^. Hence, to improve crop production, it is sensible to understand the basis for plant adaptation to drought and the implementation of strategies to apply this knowledge. The effect of drought on plants is complex as they implement many protective adaptations, among them, stomata closure is key to prevent water evaporation and to maintain hydration of cells and tissues. This in turn, is controlled by the cytosolic levels of abscisic acid (ABA), the main phytohormone involved in the adaptive responses to drought (Cutler et al., 2010; Gonzalez-Guzman et al., 2012).

Abscisic acid signaling relies on the coordinated action of the family of pyrabactin resistance 1/PYR1-like/regulatory components of ABA receptors (PYR/PYL), on the clade A subfamily of protein phosphatases type-2C (PP2C) ABA coreceptors and on the family of sucrose non-fermenting-2-related protein kinases (SnRK2; Reviewed in Rodriguez et al., 2019). The available molecular and structural information on the components of the pathway provides a detailed mechanism for ABA sensing. In the resting state, the PYR/PYL receptors are monomers or dimers and display a wide empty ABA binding cavity flanked by two highly conserved loops, named as gate loop and latch loop. In this situation, the SnRK2s are forming a stable complex with the PP2Cs that maintains the kinase inactive. The increase in the ABA concentration motivated by stress gives rise to the hormone association with PYR/PYL receptors. This includes the structural rearrangement of the gate and latch loops in the ABA binding pocket and the dissociation of dimeric receptors to provide a binding surface for the PP2Cs. The formation of PYL-ABA-PP2Cs ternary complexes releases the SnRK2s from association with the PP2Cs (Melcher et al., 2009; Miyazono et al., 2009; Nishimura et al., 2009; Santiago et al., 2009; Yin et al., 2009; Dupeux et al., 2011a,b; Yunta et al., 2011). Then, the active SnRK2s can phosphorylate downstream effectors activating transcriptional and post-transcriptional ABA mediated cell response including stomata closure. ABA-activated SnRK2s that have been previously dephosphorylated by clade A PP2Cs precise activation by phosphorylation through B2 and B3-type RAF-like MAP3Ks (Lin et al., 2020, 2021; Takahashi et al., 2020).

The knowledge derived from the characterization of the ABA pathway has provided the basis for the genetic manipulation of plants for biotechnological purposes. For instance, the overexpression of ABA receptors (Santiago et al., 2009; Mosquna et al., 2011; Pizzio et al., 2013; Gonzalez-Guzman et al., 2014; Yang et al., 2016, 2019) or the reduced expression of the PP2C coreceptors (Rubio et al., 2009; Antoni et al., 2012) diminish plant transpiration and increase drought resistance. In addition, there is a field for the structure-based rational design of novel molecules that could be used as agrochemical compounds against stress. In particular, for the development of chemical compounds that act as ABA agonists or antagonists. These molecules can modulate ABA signaling dynamically and exogenously either by enhancing drought tolerance or inhibiting ABA mediated responses in plants, respectively, (Hewage et al., 2020; Lozano-Juste et al., 2020). Major breakthroughs in this field have been the discovery of Opabactin, a potent ABA agonist for manipulating crop abiotic stress tolerance and water use (Vaidya et al., 2019) and Antabactin, a pan-receptor antagonist that disrupts ABA mediated response in *Arabidopsis* and different crop species (Vaidya et al., 2021).

The advantages and challenges of genetic and chemical approaches have been reviewed (Yang et al., 2021), however, to pursue both approaches it would be desirable to study in depth the transitions leading to the activation of the pathway. The structure-based Gate-Latch-Lock mechanism for ABA signaling proposed that the latch and gate loops undergo a well-defined conformational change from the open conformation observed in the apo form of the PYR/PYL receptors to the closed conformation adopted in the ternary PYR/PYL-ABA-PP2C complexes (Melcher et al., 2009). This description of the steps leading to ABA perception was enriched by the identification and analysis of different intermediates (Moreno-Alvero et al., 2017). Thus, Moreno-Alvero et al. (2017) identified in crop ABA receptors a novel latch-closed gate-open ABA-bound intermediate, which suggests that PYR/PYL receptors display an equilibrium state between gate-open and gate-closed in the presence of ABA. These studies also revealed that latch closure precedes ABA binding, whereas gate closure follows the ABA binding event upon conformational selection by the PP2C. This highlights the role of PP2C as ABA coreceptors as PYR/PYL receptors display an equilibrium state between gate-open and gate-closed in the ABA-bound form and consequently, the PP2C either induces the shift of this equilibrium to the gate-closed form or will selectively bind to the gate-closed conformation. An additional step should be considered for the activation of dimeric PYR/PYLs as structural studies showed that the conformational changes leading to ABA recognition for these receptors are necessarily coupled with their dissociation because the dimerization and PP2C binding interfaces overlap. The energetic cost of such dissociation gives rise to lower intrinsic affinity for ABA, which is around two orders of magnitude lower than that of the monomeric receptors (Dupeux et al., 2011b). Moreover, monomeric receptors are able to inhibit PP2C activity to some extent in absence of ABA (Hao et al., 2011).

The relevance for ABA signaling of dimeric PYLs in tomato is presumably similar to that of dimeric PYLs in *Arabidopsis* (Sun et al., 2011; Cao et al., 2013; Okamoto et al., 2013; Gonzalez-Guzman et al., 2014; Chen et al., 2016). Phylogenetic studies have shown that tomato PYLs, as those from the higher plants (Yang et al., 2020), are grouped in three subfamilies, as *Arabidopsis* PYLs, and quinabactin, which is an agonist of dimeric ABA receptors, is able to inhibit tomato seed germination and induce stress-responsive genes (Gonzalez-Guzman et al., 2014). However, genetic studies where expression of tomato PYLs is modified are still scarce (Kai et al., 2019). *In vitro* studies have demonstrated that dimeric tomato PYLs are able to inhibit the activity of clade A PP2Cs, which play a key role for ABA signaling in tomato (Gonzalez-Guzman et al., 2014; Zhang et al., 2018).

Abscisic acid receptor agonists and antagonists are important tools to achieve either hyper- or hypostimulation of ABA signaling, respectively. Chemical activation of ABA signaling can lead to enhanced drought tolerance (Cao et al., 2017; Vaidya and Cutler, 2022) whereas downregulation of ABA signaling -when water sources are not limiting—can enhance crop yield (Miao et al., 2018) or serve to stimulate seed germination (Vaidya et al., 2021).

Altogether, these data have provided a deep understanding of the pathway that can be used to rationalize the effect of engineered mutations in *Arabidopsis* and crop plants or to design molecular sensors with tailored properties. In this work, we have identified a single amino acid variation at the latch loop that classifies ABA receptors according to their oligomeric state. We have shown that a mutated dimeric PYL1 receptor from tomato (*Solanum lycopersicum*, SlPYL1) harboring this substitution renders to a receptor with enhanced ABA binding affinity and that inhibits PP2C in an ABA independent manner. The crystallographic analysis of the mutated protein has led to the identification of the basis of such behavior. Moreover, we employed a molecular dynamic based crystallographic refinement (Burnley et al., 2012) to show that the higher heterogeneity of the mutated latch loop eases the transition of the loop to a productive conformation for ABA binding. Moreover, the crystallographic analysis of the aforementioned mutant has led to the discovery of niacin as a new ABA antagonist, which blocks the latch loop by generating an intermediate and unproductive conformation of the receptor that prevents PP2C interaction.

## Materials and Methods

### Mutagenesis of SlPYL1

The SlPYL1-E151D mutant was generated from pETM11-Sl08g076960wt (Gonzalez-Guzman et al., 2014) using the QuickChange Lightning Site-Directed Mutagenesis Kit (Agilent). Briefly, we performed a PCR using forward (GTATCATTGGCGGAGATCACCGGTTGAGGAATTACC) and reverse (GGTAATTCCTCAACCGGTGATCTCCGCCAATG ATAC) primers carrying the desired mutation, followed by a DpnI digestion to restrict parental DNA. The digestion product was then transformed into *Escherichia coli* XL10 Gold cells. Positive colonies were sequenced to confirm the mutation.

### Expression of SlPYL1 Wild Type and SlPYL1 E151D and Purification of His-Tagged Proteins

*Escherichia coli* BL21 (DE3) cells were transformed with the corresponding pETM11-SlPYL1 construct and grown at 37°C to an optical density at 600 nm of 0.7 in 1 L of 2-TY medium supplemented with 50 μg/ml kanamycin. Then, 0.3 mM isopropyl-β-D-thiogalactoside (IPTG) was added to the medium, and the cells were harvested after overnight incubation at 16°C. Pellets were resuspended in lysis buffer (30 mM Tris pH 7.5, 150 mM NaCl, 1 mM DTT) and lysed by sonication in a Branson sonifier. Clear lysates were obtained after centrifugation at 20,000 *g* for 40 min, and they were purified using a 1 ml nickel-nitrilotriacetic acid agarose column, previously equilibrated with 30 mM Tris pH 7.5, 150 mM NaCl, 1 mM DTT buffer. A washing step was performed using 30 mM Tris pH 7.5, 150 mM NaCl, 20 mM imidazole, 1 mM DTT, and the His tags were cleaved by tobacco etch virus protease. The proteins were eluted using 30 mM Tris pH 7.5, 150 mM NaCl, 40 mM imidazole, 1 mM DTT buffer. Eighteen and twenty-three milligram of SlPYL1 wild-type (WT) and SlPYL1 E151D were obtained and concentrated to 4.5 and 5.8 mg/ml, respectively. These samples were injected on a HiLoad Superdex 200 16/60 column (GE Healthcare) previously equilibrated with 30 mM Tris pH 7.5, 150 mM NaCl, 1 mM DTT buffer. Fractions corresponding to dimeric SlPYL1 WT and E151D were pooled. Protein concentration was calculated using a Nanodrop spectrophotometer (Thermo Scientific) and their quality was checked by 12% SDS-PAGE (Supplementary Figure 1).

### Crystallization, Data Collection, Structure Solution, and Refinement

The crystallization protocols for all structures were identical. In summary, crystals were obtained using vapor diffusion hanging drop technique using protein at 10 mg/ml concentration, 1.6 and 1.8M ammonium sulfate pH 7.0 as precipitant solution and a protein:precipitant ratio of 1:1 and 1:2. The crystals for SlPYL1 E151D:ABA and SlPYL1:niacin complexes were obtained using the same conditions by addition of 10 mM ABA (Biosynth) or niacin (Sigma Aldrich), respectively, to crystallization drops in a ratio 1:1:0.2 or 1:2:0.2. Crystals of SlPYL1 E151D:niacin complex grew from crystallization drops in a ratio 1:1 whereas crystals of SlPYL1 E151D apo protein grew from a ratio 1:2.

Crystals were cryoprotected in the crystallization solution containing 25–30% glycerol, mounted on a fiber loop and flash-frozen in liquid nitrogen. A complete diffraction data set was collected (see details in Supplementary Table 1). Diffraction data were processed with XDS (Kabsch, 2010) and merged with AIMLESS from the CCP4 package (Collaborative Computational Project, Number 4, 1994; Winn et al., 2011). We employed the crystal structures of SlPYL1 (PDB code 5MOA) and SlPYL1 in complex with ABA (PDB code 5MOB; Moreno-Alvero et al., 2017) to phase the diffraction data of crystal of the apo form of SlPYL1 E151D, the ABA and Niacin complexes and the SlPYL1 Niacin complex as the crystals were nearly isomorphous. Several cycles of restrained refinement with PHENIX (Adams et al., 2010) and iterative model building with COOT (Emsley and Cowtan, 2004) were required to obtain the final models where the waters were also modeled. Data collection, data processing and model refinement statistics are summarized in Supplementary Table 1. The stereochemistry of the models was verified with MolProbity and figures of molecular models were produced using PyMOL (The PyMOL Molecular Graphics System, Version 1.6.0.0 Schrödinger, LLC).

### Modeling of Dynamics in SlPYL1 and SlPYL1 E151D Crystal Structures

To extract dynamical details from the X-ray data, the refined coordinates of native SlPYL1 were first re-refined using PHENIX (Adams et al., 2010). These coordinates and those of SlPYL1 E151D were used as input models for a time-averaged molecular dynamics refinement as implemented in the Phenix.ensemble-refinement routine, which was performed as described previously (Burnley et al., 2012). The ensemble refinement method performs molecular dynamic simulations to sample local atomic fluctuations of SlPYL1 in the crystal separating large-scale motions attributable to the rigid movement of secondary structures or lattice distortions. The method prevents over-fitting of the data by the restriction of the number of structures modeled to 50 models. The calculated models for the apo forms of the SlPYL1 and SlPYL1 E151 shows improved fit to the X-ray data as R-free improved by 2.2 and 3.2 percentage points. Prior to the simulations, we approximated the large-scale disorder by an overall TLS model derived from the atomic *B*-factors of the refined single structure. The simulations were run at an effective temperature of 300 K for the protein atoms. The parameters p-tls, which is the percentage of atoms included in TLS-fitting, was optimized to 90%, 0.9, in a grid search to obtain the minimum R-free after the refinement.

### PP2C Activity Assays

Phosphatase activity was measured using p-nitrophenyl phosphate (pNPP) as a substrate. The assays were performed in quadruplicates in a 100 μl solution containing 25 mM Tris–HCl pH 7.5, 75 mM NaCl, 0.5 mM DTT, 1 mM MnCl2, and 25 mM pNPP. The assays included molar relations of phosphatase:receptor of 1:2 or 1:4 (0.5 μM ΔN-HAB1 and 1 or 2 μM SlPYL1 WT or mutant), and the indicated concentration of ABA or nicotinic acid. We chose the PP2C from *Arabidopsis* HAB1 because successful procedures to determine the half maximal inhibitory concentrations (IC_50_) as ABA receptors are able to inhibit PP2Cs from different plant species and successful protocols using HAB1 have been previously reported (Moreno-Alvero et al., 2017; Lozano-Juste et al., 2021). The activity was recorded with a FLUOstar Omega plate reader at 405 nm every 5 min over 20 min, and the activity obtained after 20 min was indicated in the graphs.

### Thermal Shift Assay

Label-free thermal shift assays with SlPYL1 WT and SlPYL1 E151D were performed using Tycho NT. 6 (NanoTemper Technologies). Assays were performed at 6 μM protein in 30 mM Tris–HCl, pH 7.5, 150 mM NaCl, 1 mM DTT and run in quadruplicates in Tycho NT.6 capillaries (Cat no. TY-C001; NanoTemper Technologies), using final ABA concentrations of 0.067, 0.25, 1, and 4 mM. Intrinsic fluorescence was recorded at 330 and 350 nm while heating the sample from 35 to 95°C at a rate of 30°C/min. Fluorescence ratio (350/330 nm) and inflection temperature (Ti) were calculated by Tycho NT. 6.

### Size Exclusion Chromatography

Refractive index measurements were carried out at different protein concentrations. 50 μl of protein solutions with 60, 6, or 0.6 μg of SlPYL1 WT or mutant in 30 mM Tris–HCl, pH 7.5, 150 mM NaCl, 1 mM DTT buffer were loaded on a Superdex 30 Increase 3.2/300 column (GE Healthcare). The refractive index of the samples was recorded over time using a Shimadzu LC-20AD Prominence HPLC Pump (Shimadzu Scientific Instruments).

### Plants

Approximately 25 seeds (two replicates per experiment) of *Arabidopsis thaliana* Col-0 seeds were sown on 24-multiwell plates containing MS medium, which was mock- or ABA-supplemented at 0.125 or 0.250 μM concentration. Increasing concentrations (from 0.5 to 10 μM) of nicotinic acid were added in mock- or ABA-supplemented plates. Seedling establishment and early seedling growth was scored at 5 and 9 days after sowing. Root length was scored at 5 days after sowing.

## Results

### Glutamic to Aspartic Acid Substitution at Latch Loop of SlPYL1 Enhances Receptor Ability to Inhibit the Phosphatase HAB1

The comparison of the available structures of PYR/PYL receptors showed that latch closure is a prerequisite for ABA binding, in turn for gate closure and for the subsequent phosphatase inhibition (Melcher et al., 2009; Moreno-Alvero et al., 2017; Rodriguez et al., 2019). This conformational rearrangement involves the concerted movement of a conserved Glu/Asp-His-Arg motif at the latch to make the binding site accessible to ABA and to define its binding pocket. Interestingly, while the His-Arg sequence is conserved among the whole family of PYR/PYL receptors, the substitution Glu to Asp differentiates the group of the dimeric receptors from the monomeric ones (Supplementary Figure 2). Accordingly, the amino acid sequence alignment of tomato receptors showed that the corresponding residue in monomeric PYLs of tomato is also Asp (Gonzalez-Guzman et al., 2014). To investigate the relevance of this amino acid on the receptor function, we generated a mutated version of SlPYL1 harboring a Glu 151 to Asp substitution (SlPYL1 E151D) at the latch and studied its biochemical properties and compared them with those of the native protein.

**FIGURE 1:**
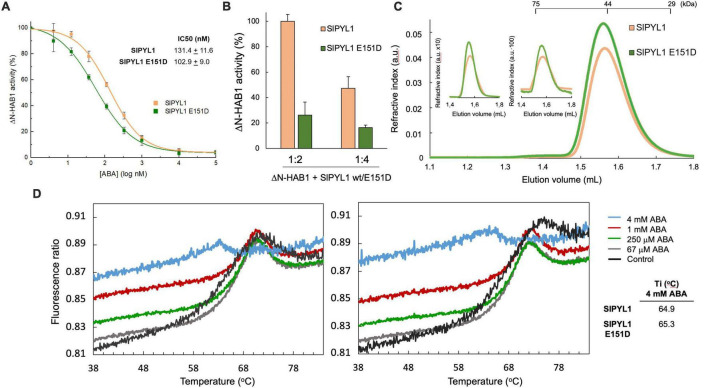
Biochemical properties of SlPYL1 E151D. **(A)** Determination of the IC50 for inhibition of HAB1 by ABA and SlPYL1 or SlPYL1 E151D at 1:2 molar proportion of HAB1:SlPYL1. **(B)** HAB1 activity in the presence of molar ratios of HAB1:SlPYL1 or HAB1:SlPYL1 E151D of 1:2 and 1:4. 100% of activity is referred to 1:2 HAB1:SlPYL1wt. **(C)** Elution profile of SlPYL1 and SlPYL1 E151D, loading 60 (main profile), 6 or 0.6 microg of protein (inset). The positions of the molecular weight markers are indicated. **(D)** Thermal denaturation profiles of (left) wild-type and (right) mutant SlPYL1 E151D proteins (6 mM) in the absence or presence of increasing ABA concentrations (legend). The inflection temperature at 4 mM ABA is indicated.

We first analyzed the PP2C inhibitory activity of the WT and mutated receptor *in vitro*. Our data showed that IC_50_ value of ABA using SlPYL1 E151D was 103 ± 9 nM which is significantly smaller than that calculated using WT protein 131 ± 11 nM (Figure 1A). In addition, we examined the inhibitory effect of both WT and mutant receptors in absence of ABA with HAB1:receptor ratios of 1:2 and 1:4. Interestingly, we observed a higher reduction in phosphatase activity using SlPYL1 E151D when compared to SlPYL1 (Figure 1B).

The enhanced activity of the mutated receptor suggests that the Asp 151 residue might be involved in the dissociation of the dimeric structure despite it is located apart from the oligomerization interface. Hence, we investigated the oligomeric state of the WT and SlPYL1 E151D mutant protein by Size-Exclusion Chromatography (SEC). We performed a series of SEC experiments varying the protein concentration injected from 48 to 0.48 μM revealing that the mutation does not affect the oligomeric state of the apo form of both proteins as they form dimers in solution at all the assayed concentrations (Figure 1C).

Protein stability and biological activity are usually coupled when comparing native and mutated enzymatic systems (Rodriguez-Larrea et al., 2010; Mesa-Torres et al., 2014). We therefore studied the effect of the amino acid substitution on the stability of the apo and ABA bound forms of the protein using a label-free thermal shift assay (nano-Differential Scanning Fluorimetry, nanoDSF, Tycho NT. 6, NanoTemper Technologies). Using this technique, the intrinsic fluorescence of the receptors without or at increased concentrations of free ABA were recorded at 330 and 350 nm while heating the sample from 35 to 95°C. The variation of the ratio of fluorescence (350/330 nm) versus the Ti usually follows a sigmoidal curve which is related to the cooperative thermal denaturation of the protein. The analysis of this curve provides an inflection Ti that reports on the stability of the protein or on the formation of a complex with a ligand if a binding event occurs. However, in absence of ABA, complex denaturation profiles of both WT and SlPYL1 E151D proteins were observed. Interestingly, this complexity is reduced at increased ABA concentrations to a point at 4 mM ABA in which a simple sigmoidal transition is observed. As the dimeric receptors tend to dissociate upon ABA binding (Dupeux et al., 2011b), this suggests that the transition from native to unfolded protein at low ABA concentration occurs through two independent events, the dissociation of the dimeric species and the denaturation of the monomeric protein (Figure 1D). The complex nature of the denaturation profiles obtained without or at low concentrations of ABA hinders the calculation of Ti, however, SlPYL1 E151D protein displays larger Ti than the WT in the presence of 4 mM ABA, showing that the Asp side chain is involved in the stabilization of the protein.

### The Structure SlPYL1 E151D Provides the Basis for Its Enhanced Abscisic Acid Dependent Activity

**FIGURE 2:**
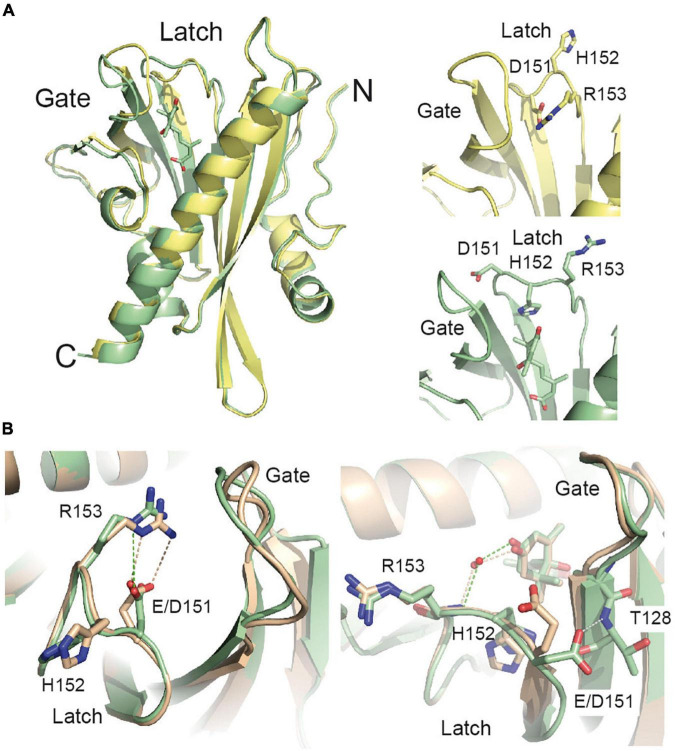
The structure of the SlPYL1 E151D. (**A**; Right) Superposition of the structures of the apo and ABA bound SlPYL1 E151D (yellow and green, respectively) together with a detail of the residues conforming the Glu/Asp-His-Arg motif (left). The view faces the dimerization interface. The latch and gate loops are highlighted. **(B)** Superposition of a section of the structures of the apo and ABA bound SlPYL1 E151D (green; upper and lower panel, respectively) and SlPY1 in complex with ABA [wheat; Protein Data Bank (PDB) codes 5MOA and 5MOB, respectively]. Hydrogen bonds are represented as dashed lines.

The structure of the SlPYL1 and other ABA receptors revealed that Glu 151 is located in the interface of the gate and latch loops (Moreno-Alvero et al., 2017), hence, we reasoned that E151D mutation might be affecting the conformational rearrangements leading the receptor activation. To investigate this question and to determine the structural basis of the effect of E151D mutation on ABA perception, we underwent crystallographic studies (Figure 2, Supplementary Table 1, and section “Materials and Methods”). The overall structures of the apo and ABA bound forms of SlPYL1 E151D are nearly identical to that of WT protein with the differences confined to the area of the mutated residue. The Root Mean Squared Deviation for 191 Calpha atoms are of 0.33A and 0.20A for the apo and ABA bound forms, respectively.

Previous crystallographic data on native SlPYL1 showed that the transition from the closed to the open conformation of the latch included a large rearrangement of Glu 151 (Moreno-Alvero et al., 2017). In this structure, the apo form showed that Glu 151 and Arg 153 established a salt bridge that blocked the ABA binding pocket while, in the ABA bound form, Glu 151 pointed to the solvent enabling ABA binding and subsequent gate closure (Moreno-Alvero et al., 2017; Figure 2B). Instead, the analysis of the apo form of SlPYL1 E151D structure revealed that the shortening of the Glu 151 side chain to Asp disrupts the geometry of the salt bridge with Arg 153, weakening this interaction and favoring the transition to an active receptor. In addition, the ABA bound structure shows that Asp 151 carboxylate is hydrogen bonded to the NH of Thr 128 at the C-terminal end of the gate loop. This hydrogen bond is not present in the native structure and might account for the increased stability of the mutant protein in complex with ABA with respect to that observed for the native protein (Figure 1D). Interestingly, the ABA bound forms of the receptors display a wider aperture at the binding site that the apo forms, this feature is also observed for citrus PYL1 and other crop receptors (Moreno-Alvero et al., 2017; Figure 2).

**FIGURE 3:**
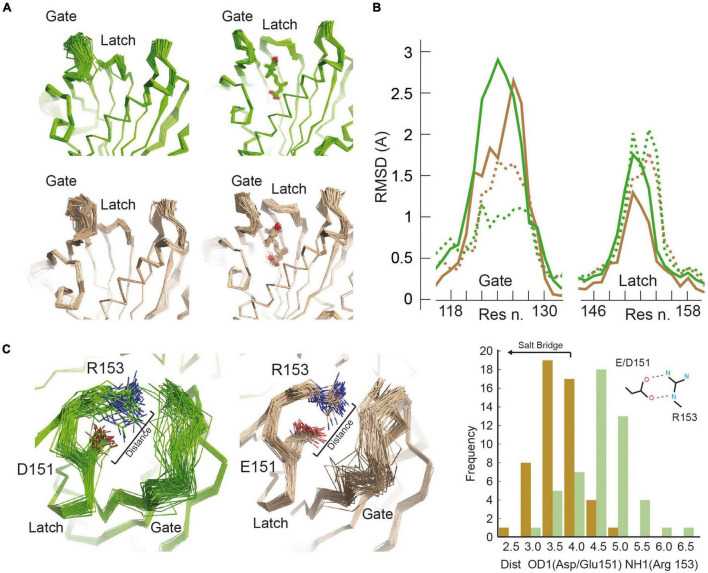
Molecular motions of SlPYL1 E151D. **(A)** Overview of main chain dynamics of gate and latch in ensemble structures of the apo and ABA bound forms of SlPYL1 E151D (green) and SlPYL1 (wheat). **(B)** Main chain atomic root mean squared fluctuations at gate and latch of ensemble models of apo and ABA SlPYL1 E151D (solid and dashed green lines, respectively) and apo and ABA SlPYL1 (solid and dashed wheat lines, respectively). (**C**; Left) Side chain dynamics of residues conforming the salt bridge between Glu/Asp 151 and Arg 153 of the apo forms of SlPYL1 E151D (green) and SlPYL1 (wheat). (Right) Panel showing the corresponding minimum distance between Asp (green) or Glu 151 (wheat) carboxylate oxygen atoms and Arg 153 guanidinium nitrogen atoms distribution.

These results highlight the importance of the dynamical aspects on ABA perception and pathway activation. Thus, to gain insights into these issues, we performed a molecular dynamic based ensemble refinement restrained by crystallographic data (Adams et al., 2010; Figure 3A, Supplementary Table 1, and section “Materials and Methods”). This protocol provides a realistic picture of the molecular fluctuations of a protein within a crystal by assembling multiple models of its structure (Burnley et al., 2012). Hence, in combination with the traditional crystallographic refinement, the protocol enables the investigation of the consequences of the E151D mutation on the dynamic properties of gate and latch loops.

The analysis of the superimposition and the root mean square deviation (RMSD) per residue along the 50 models that result from the ensemble refinement illustrates the dynamics in SlPYL1 and SlPYL1 E151D crystal structures. The increased RMSD of the gate and latch loops in the apo forms of SlPYL1 E151D with respect to those of SlPYL1 (Figures 3A,B) shows that mutated protein displays higher conformational heterogeneity at these loops. In addition, it reveals that the native structure displays a well-ordered salt bridge between Glu 151 and Arg 153 blocking ABA binding while the mutant protein displays high flexibility for Asp 151 and Arg 153 (Figure 3C), thus suggesting that the Asp151 mutation might ease the large transition – of the latch for a productive ABA recognition. Interestingly, there is a reduction of the RMSD as a result of ABA binding, that indicates that hormone binding stabilizes the entire gate loop despite it does not make any direct contact with ABA (Figures 3A,B). However, the degree of such stabilization is higher in the mutant protein.

### The Crystallographic Studies on SlPYL1 E151D Unravels Niacin as Antagonist Molecule of Abscisic Acid Activity

**FIGURE 4:**
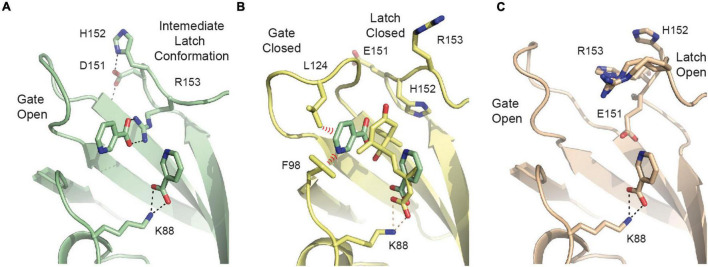
The SlPY1 E151D Niacin complex. **(A)** The network of hydrogen bond interactions with niacin in the complex with SlPYL1 E151D. **(B)** The overlay of the niacin molecules from the complex with SlPYL1 E151D onto the CsPYL1-ABA-HAB1 complex, residues are labeled according to SlPYL1 sequence. Red dashed symbols highlight hypothetical short unfavorable clashes between niacin and residues of the gate loop in closed conformation. **(C)** The network of hydrogen bond interactions with niacin in the complex with SLPYL1.

The crystals of SlPYL1 E151D grown at 1:1 protein:precipitant ratio without ABA displayed two residual blobs of density at the ABA site that we interpreted chemically as two nicotinic acid molecules, also called niacin, product of bacterial expression (Figure 4A and Supplementary Figure 3A). Interestingly, the ligand molecules mimic the position and some of the interactions observed for ABA in the CsPYL1-ABA-HAB1 ternary complex (Moreno-Alvero et al., 2017; Figure 4B). The first site is interacting with the α amino group NZ of Lys 88 and mimics the carboxylate moiety of ABA, while the second interacts with the indole moiety Arg 153. In this situation, Asp 151 adopts the ABA bound conformation while His 152 points toward solvent as it is observed in the apo crystal form. Thus, this conformation represents an intermediate between that observed for the apo and ABA bound structures. Remarkably, Asp 151 is not interacting with niacin, rather it is involved in the stabilization of the conformation of the loop via side chain hydrogen bonds to NH of Thr 128 and NE of His 152.

Attempts to reproduce the crystallization conditions yielding to a niacin complex with the WT protein were unsuccessful, hence we grew crystals of SlPYL1 with a solution containing 1.0 mM niacin and solved the crystal structure to investigate the role of Glu to Asp 151 substitution in the loop conformation. Our data show that the WT protein interacts solely with a molecule of niacin at ABA carboxylate site which is interacting with NZ of Lys 88 (Figure 4C and Supplementary Figure 3B). These data suggest that formation of the complex with two molecules of niacin would be a consequence of the higher flexibility of the mutated latch loop with respect to that of the WT structure (Figure 3).

Our crystallographic data shows that the interaction of niacin at the ABA carboxylate site both in the native and in the mutant protein, would compete with ABA for the binding site and might provide the basis for a universal ABA antagonist molecule. Moreover, niacin hinders gate and latch closure of the SlPYL1 E151D mutant protein (Figure 4B), hence, we reasoned that as monomeric receptors naturally harbor Asp at the Glu/Asp-His-Arg latch motif, niacin may represent an effective ABA antagonist of this family of receptors.

**FIGURE 5:**
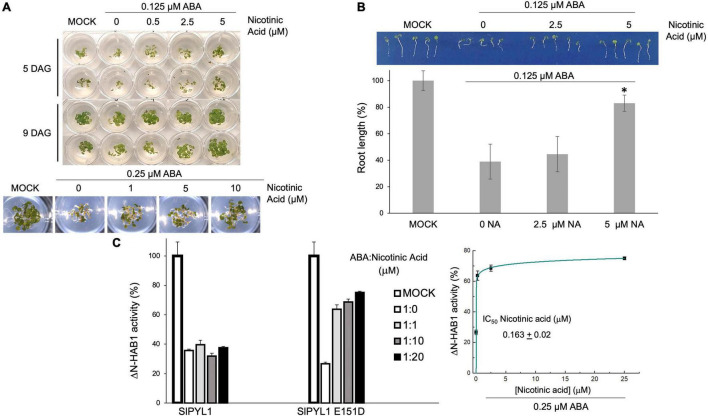
ABA-mediated inhibition of seedling establishment is counteracted by increasing concentrations of nicotinic acid. **(A)** Approximately 25 seeds (two replicates per experiment) of Arabidopsis thaliana Col-0 seeds were sown on 24-multiwell plates lacking (MOCK control) or supplemented with 0.125 μM ABA. Increasing concentrations (0.5, 2.5, and 5 μM) of nicotinic acid were added in those wells supplemented with 0.125 μM ABA. Seedling establishment and early seedling growth was scored at 5 and 9 days after sowing. **(B)** Root length was scored at 5 days after sowing. *p < 0.05. **(C)** ABA-antagonist activity of nicotinic acid. Increasing concentrations (0.25, 2.5, and 5 μM) of nicotinic acid were added to the reaction mixture supplemented with 0.25 μM ABA.

To test whether niacin can antagonize the effects of ABA *in vivo*, we performed *Arabidopsis* seed germination/seedling establishment assays. ABA inhibits seedling establishment and early seedling growth; therefore, we analyzed whether different concentrations of niacin coapplied with ABA could restore seed establishment of *Arabidopsis*. Figure 5A shows that increasing concentrations of niacin relieve the inhibition of seedling establishment and early seedling growth when coapplied with ABA. This was apparent at concentrations between 20- and 40-fold higher than that of ABA. In particular, Figure 5B shows that root length was restored when 5 μM Niacin was coapplied with 0.125 μM ABA. Thus, this revealed niacin as an ABA antagonist *in vivo*.

To investigate whether the antagonistic effect of niacin was mediated by the binding of the compound to the carboxylate site or to the latch loop of the receptor, we examined the ability of niacin to reverse ABA mediated PP2C inhibition using WT and mutant SlPYL1. Our data showed that niacin produces a clear protective effect against ABA enabling a recovery of HAB1 activity even at equimolecular concentrations of ABA and niacin in the presence of SlPYL1 E151D (Figure 5C). Conversely, no effect was observed when assaying the WT protein. This shows that antagonistic effect of niacin requires the presence of Asp 151 at the latch loop.

## Discussion

The design of an ABA receptor of an economically valuable crop specie to be ABA independent or to display an exacerbated response to ABA represents a biotechnological opportunity to produce genetically modified plant varieties with enhanced performance in drought stress situations. Tomato ABA receptors have been characterized (Gonzalez-Guzman et al., 2014) and, as those from *Arabidopsis* (Dupeux et al., 2011b), they are classified as monomeric or dimeric according to their oligomeric state. As the monomeric receptors display higher ABA binding affinities than the dimeric ones and are able to form low affinity ABA independent complexes with PP2Cs, it is sensible to identify those residues that are conserved among the members of each class and analyze if they are involved in the receptor activity. Indeed, previous work led to the identification of a single point mutation in the dimerization interface of PYR1 ABA receptor that enhances the ABA binding affinity one order of magnitude despite this mutation is localized apart from the hormone binding site (Dupeux et al., 2011b). To investigate this issue, we have selected as a model SlPYL1 which is a tomato dimeric receptor with known structure (Moreno-Alvero et al., 2017) that is involved in the induction of stress responsive genes and drought tolerance (Gonzalez-Guzman et al., 2014).

The sequence analysis of SlPYL1 and those ABA receptors from *Arabidopsis* led to the identification of Glu 151 which is a unique position in the alignment as it is conserved among the dimeric receptors and that is replaced by Asp in the monomeric ones. Interestingly, the analysis of the structure of SlPYL1 shows that Glu 151 is not involved in the dimerization interface or interacting with ABA; rather, it is located in the latch loop (Figure 2). Hence, to investigate the effect of this substitution, we prepared a SlPYL1 E151D protein and performed biochemical and structural studies. As expected, we did not observe any effect on the oligomeric properties of the mutated receptor (Figure 1C). However, our data showed that Glu 151 to Asp enhances the ability of SlPYL1 to inhibit the phosphatase HAB1 in an ABA dependent and independent manner when compared with the WT protein (Figures 1A,B). This suggests that the mutation is stimulating the conformational rearrangements of the latch loop required for ABA recognition. Indeed, our structural studies corroborated that the mutated latch loop displays higher heterogeneity than the WT, due to the disruption of a salt bridge between Glu 151 and Arg 153 which is capping the entrance to the ABA binding site in the apo form of the receptor (Figures 2, 3). In addition, the analysis of the ABA bound structures of SlPYL1 E151D and SlPYL1 showed that Glu 151 replacement to Asp leads to the formation of an additional hydrogen bond linking Asp 151 at the latch and Thr 110 at the gate (Figure 2B). This would explain our data showing that the ABA bound form of SlPYL1 E151D displays increased stability as compared with that of SlPYL1 (Figure 1D).

**FIGURE 6:**
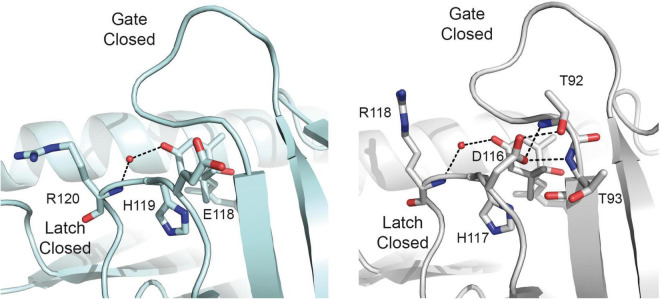
Asp at the latch contributes to the stabilization of the closed conformation of the gate. A section of the structures of dimeric AtPYL2-ABA (left) and monomeric AtPYL9-ABA (right). Selected hydrogen bonds are highlighted as dashed lines.

Altogether, the joined analysis of the biochemical and crystallographic data suggests that the weakening of the capping salt bridge in the apo structure of the mutant protein together with the formation of an additional hydrogen bond linking Asp 151 and Thr 110 in the ABA bound structure shift the equilibrium from the apo form to the active form of the mutant receptor. This would not be sufficient to dissociate the dimer, but would enhance the ABA dependent and independent binding activity. This suggests that the presence of Glu side chain in the Glu/Asp-His-Arg latch motive of dimeric receptors contributes to increase their ABA dependent activity with respect to the monomeric ones in addition to the unfavorable contribution derived from dimer dissociation (Dupeux et al., 2011b; Okamoto et al., 2013). To explore further whether the Glu to Asp substitution stabilizes a productive gate conformation, we analyzed the crystal structures of the ABA forms of the *Arabidopsis* receptors available in the literature. These structures display a productive gate conformation despite they are not in complex with a PP2C. Our study indicates that while monomeric AtPYL9 shows that Asp 117 is hydrogen bonded to Thr 92 and Thr 93 NH at the gate (Zhang et al., 2013), the Glu 188 side chain of the dimeric AtPYL2 is fully exposed to the solvent (Yin et al., 2009; Figure 6). Identical situation can be observed for monomeric AtPYL10 (PDB code: 3R6P) and for dimeric AtPYR1 (Nishimura et al., 2009), AtPYL1 (Miyazono et al., 2009), and AtPYL3 (Zhang et al., 2012), respectively. This data indicates that as observed in SlPYL1 E151D, the Asp side chain at the latch loop contributes to the stabilization of a productive conformation of the monomeric receptors.

SlPYL1 E151D displays an ABA independent phosphatase inhibitory activity (Figure 1C). Other approaches have been employed to identify specific substitutions to generate activated dimeric receptors in the absence of ABA. These include the mutation of PYL4 residues located in the interface between receptor and PP2C (Pizzio et al., 2013) or to perform random site-saturation mutagenesis of AtPYR1 receptor (Mosquna et al., 2011). The latter was successful for the identification of combinations of three- and four-point mutations that led to a constitutively active receptor adopting gate closed conformation. Interestingly, while these mutations were located at the dimerization interface, Glu 151 to Asp mutation represents an additional hot spot as it is located in the latch loop. Altogether, our work illustrates that the high-resolution structural information of PYR/PYL ABA receptors may be employed to rationally design a receptor with ABA dependent and independent enhanced properties.

The crystallographic analysis of SlPYL1 E151D led to the identification of niacin as an ABA antagonist molecule. Our data showed that two molecules of niacin were blocking the ABA binding site and stabilizing the latch loop in an unproductive conformation that would hinder gate closure. Remarkably, the purification protocol of SlPYL1 E151D (section “Materials and Methods”) includes a buffer exchange step after Ni^2+^ affinity chromatography and a slow size exclusion chromatographic step on a 120 ml Superdex 200 column, thus indicating that the binding of niacin to SlPYL1 E151D displays slow kinetics. However, we produced an estimation of niacin potence as antagonist using HAB1 phosphatase recovery assay in the presence of 0.25 μM ABA. Our data shows that niacin reverses ABA-mediated HAB1 phosphatase inhibition using SlPYL1 E151D in the sub-micromolar range (Figure 5C). However, in comparison to other antagonists, niacin is 100-fold less potent than the best-known ABA antagonist molecule (Vaidya et al., 2021).

The effect of niacin *in vivo* was investigated using seed germination/seedling establishment assays. These experiments rely on the inhibitory effect of ABA on germination of *Arabidopsis* seeds. Our data showed that although niacin reverted this inhibitory effect when coapplied with ABA, this activity required a high molar excess of compound to ABA to be observed (Figure 4). However, Niacin is a precursor of Nicotinamide adenine dinucleotide (NAD), which has been documented in plants to function in response to environmental stresses including pathogen infections (Alferez et al., 2018). Plant levels of NAD and its precursors are in the millimolar or sub-millimolar range (Zhang and Mou, 2009); therefore, the observed effect with micromolar niacin concentration might be physiological.

To investigate the molecular basis of niacin activity, we performed SlPYL1 and SlPYL1 E151D mediated PP2C inhibition assays. Our data showed that while niacin is able to reverse ABA mediated HAB1 phosphatase inhibition using SlPYL1 E151D, it does not display any effect in presence of WT protein. This indicates that despite Asp 151 is not directly interacting with niacin it is required for its antagonistic activity. Consequently, the observed *in vivo* activity might be mediated by monomeric receptors as they harbor Asp at the Glu/Asp-His-Arg latch motif. This is relevant as niacin might be useful for dissecting physiological roles of monomeric ABA receptors *in vivo*. In addition, as intracellular basal ABA levels are sufficient to mediate ABA response of monomeric receptors (Tischer et al., 2017), it is tempted to speculate that the physiological basal of niacin might be modulating the ABA affinity of those monomeric receptors and consequently their activity in response to stress situations. Besides, niacin might constitute a natural precursor of new environmentally friendly family of agrochemical compounds application to fight against drought stress.

## Data Availability Statement

The datasets presented in this study can be found in online repositories. The names of the repository/repositories and accession number(s) can be found below: http://www.wwpdb.org/, 7Z1P; http://www.wwpdb.org/, 7Z1Q; http://www.wwpdb.org/, 7Z1R; and http://www.wwpdb.org/, 7Z1S.

## Author Contributions

AA: conceptualization, formal analysis, investigation, and writing–original draft preparation. LI and AA: crystallization and structural work. MR-M, MD-M, and JB: protein expression and purification. MR-M: activity and characterization assays. JO-C, AC, JL-J, and PR: plant assays. All authors contributed to the manuscript version and editing and approved the submitted version of the manuscript.

## Conflict of Interest

The authors declare that the research was conducted in the absence of any commercial or financial relationships that could be construed as a potential conflict of interest.

## Publisher’s Note

All claims expressed in this article are solely those of the authors and do not necessarily represent those of their affiliated organizations, or those of the publisher, the editors and the reviewers. Any product that may be evaluated in this article, or claim that may be made by its manufacturer, is not guaranteed or endorsed by the publisher.

## Footnotes

1
https://www.fao.org/sustainability/news/detail/en/c/1274219/
