# Supplementary material for: Structure-Based Modulation of the Ligand Sensitivity of a Tomato Dimeric Abscisic Acid Receptor Through a Glu to Asp Mutation in the Latch Loop
Source: Front Plant Sci. 2022 Jun 6;13:884029. doi: 10.3389/fpls.2022.884029 (PMC9207482; doi:10.3389/fpls.2022.884029)
Supplement: Supplementary file 1 [file Data_Sheet_1.pdf]

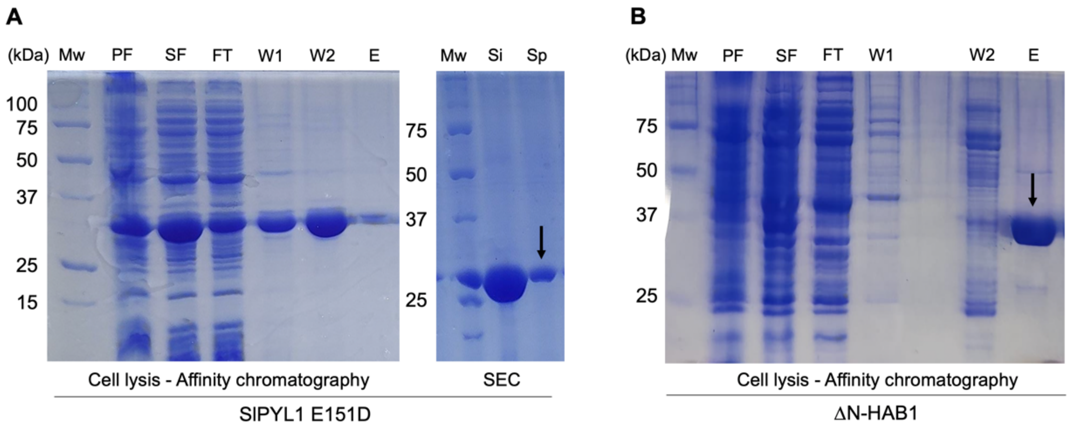

Supplementary Figure 1. 12% SDS-PAGE protein profiles of (A) SlPYL1 E151D and (B) DN-HAB1 after the indicated purification step. Mw: molecular weight marker; PF: precipitated fraction; SF: soluble fraction; FT: flowthrough; W1: wash 1; W2: wash 2; E: elution; Si: Size exclusion Chromatography (SEC) input; Sp: final protein pool after SEC. Arrows indicate the final protein quality for each sample.

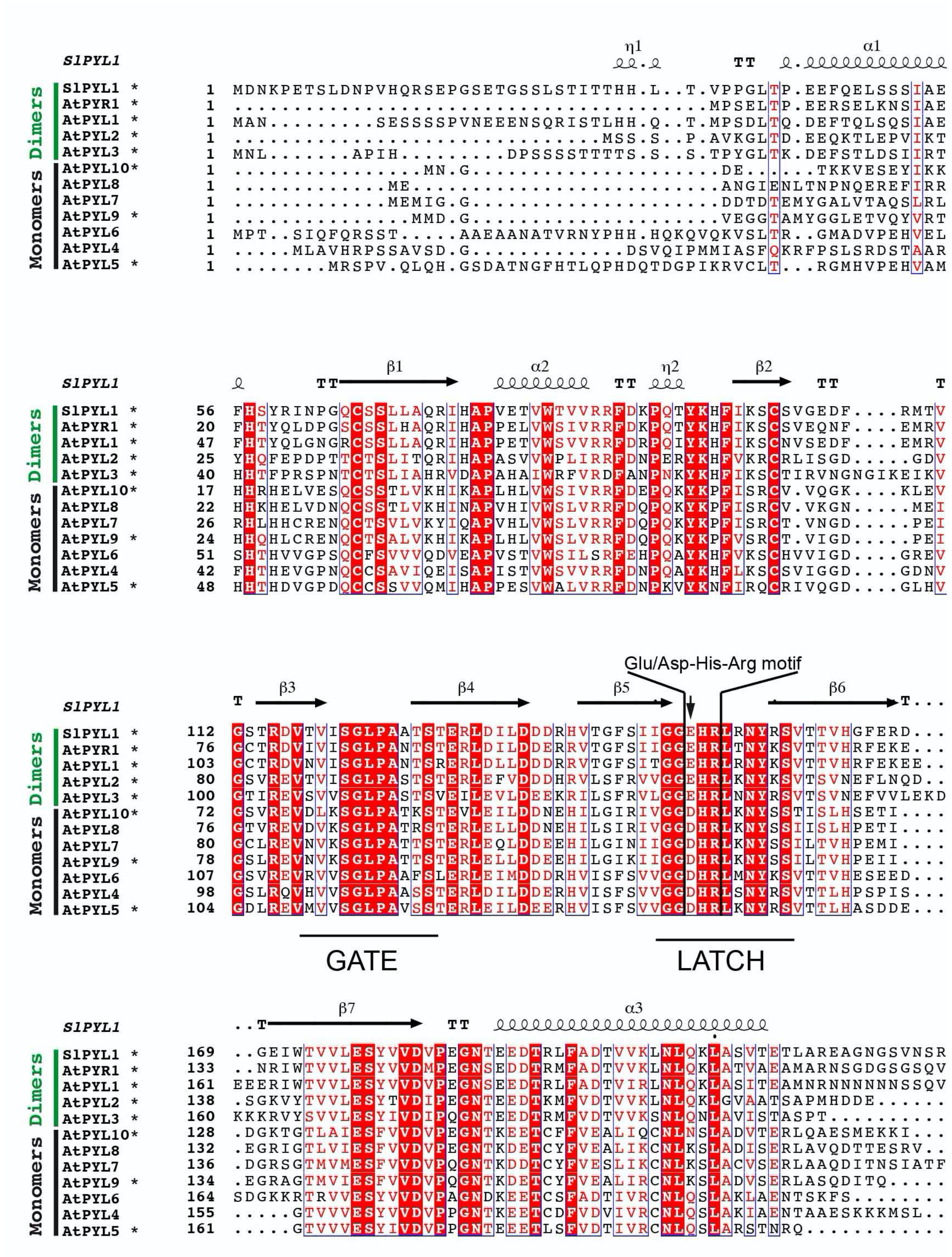

Supplementary Figure 2. The Glu/Asp-His-Arg motif differentiates the group of the dimeric receptors from the monomeric ones. Amino acid sequence alignment of SlPYL1 and a representative group of Arabidopsis PYR/PYL receptors. Proteins of known structure are marked with an asterisk. Latch and gate loops are indicated. The secondary structural elements of SlPYL1 are indicated as horizonal arrows and “T” stands for residues involved in a β-turn. Glu 151 Asp point mutation is indicated with vertical arrow.

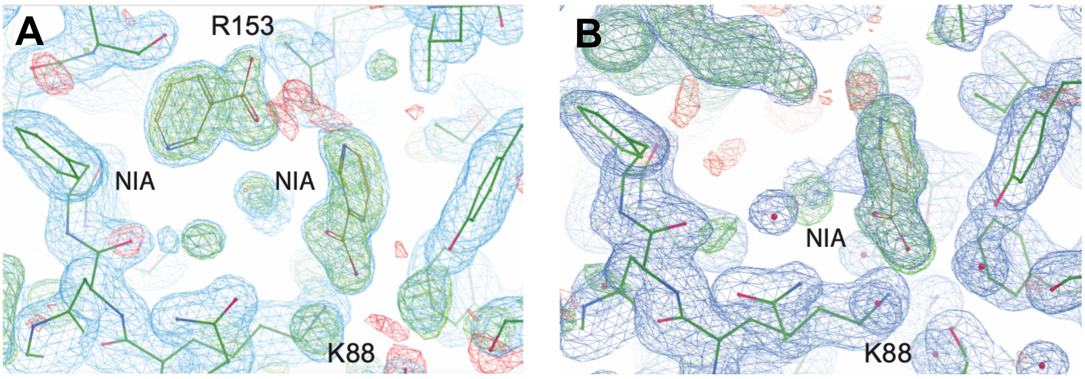

Supplementary Figure 3. A section of electron density maps at the binding pocket of SlPYL1 E151D (left) and SlPYL1 (Right). Maps were calculated after molecular replacement (using 5moa as a reference model), and several rounds of refinement in the absence of ligands plus modeling of the side chains using Coot. Shown in blue are the 2Fo-Fc and in green/red are the Fo-Fc density maps using a sigma level of 1 and 3 respectively. The latch for the SlPYL1 niacin complex was omitted at this point of refinement to build a disorder model; therefore, the green density in the upper left corner belongs to the position of the latch in addition to a difficult-to-model density near the element of symmetry between the protein monomers.
