# Supplementary material for: Structure-Based Modulation of the Ligand Sensitivity of a Tomato Dimeric Abscisic Acid Receptor Through a Glu to Asp Mutation in the Latch Loop
Source: Front Plant Sci. 2022 Jun 6;13:884029. doi: 10.3389/fpls.2022.884029 (PMC9207482; doi:10.3389/fpls.2022.884029)
Supplement: Supplementary file 2 [file Table_1.docx]

**Table S1: Diffraction data collection and refinement statistics for SlPYL1 E151D, SlPYL1 E151D:Niacin, SlPYL1 E151D:ABA and SlPYL1 Niacin (output from Phenix generate table)**

| **Data Collection** | | | | |
| --- | --- | --- | --- | --- |
| Data set | **SlPYL1 E151D** | **SlPYL1 E151D:Niacin** | **SlPYL1 E151D:ABA** | **SlPYL1 Niacin** |
| Crystal System, Space Group | Trigonal, P3_2_21 | Trigonal, P3_2_21 | Trigonal, P3_2_21 | Trigonal, P3_2_21 |
| Cell dimensions | | | | |
| a, b, c (Å) | 89.97, 89.97, 51.77 | 87.16, 87.16, 55.28 | 87.81, 87.81, 55.18 | 86.67, 86.67, 55.90 |
| α,β,γ (º) | 90, 90, 120 | 90, 90, 120 | 90, 90, 120 | 90, 90, 120 |
| Wavelength (Å) | 0.979261 | 0.979260 | 0.979182 | 0.979260 |
| Total refections | 360339 (35114) | 272830 (18110) | 506414 (49151) | 411962 (39697) |
| ^#^R_pim_ (%) | 1.47 (49.77) | 1.43 (39.54) | 1.34 (23.50) | 1.25 (92.31) |
| ^+^CC_1/2_ (%) | 100 (87.9) | 99.9 (79) | 100 (95.8) | 100 (51.5) |
| <I/σ(I)> | 23.84 (1.72) | 31.29 (1.90) | 25.46 (2.95) | 27.19 (0.68) |
| Completeness (%) | 99.76 (99.16) | 99.84 (99.05) | 99.83 (99.57) | 99.60 (96.92) |
| Wilson B-factor | 38.75 | 29.25 | 28.06 | 36.77 |
| Multiplicity | 16.4 (16.3) | 9.8 (6.6) | 15.5 (15.3) | 14.0 (13.8) |
|  | | | | |
| **Refinement** | | | | |
| Resolution range (Å) | 43.12 - 1.82 (1.89 - 1.82) | 44.6 - 1.68 (1.74 - 1.68) | 38.02 - 1.60 (1.66 - 1.60) | 43.34 - 1.65 (1.71 - 1.65) |
| Ref. used in refinement | 21962 (2149) | 27747 (2726) | 32621 (3216) | 29454 (2883) |
| R_work_/R_free_ (%) | 20.94 / 24.17  (42.40 / 47.92) | 19.98 / 22.02  (38.98 / 49.34) | 19.16 / 21.51  (31.71 / 39.65) | 22.81 / 26.70  (45.76 / 47.85) |
| Asymmetric unit content | | | | |
| Protein residues | 192 | 191 | 193 | 191 |
| Ligand/NIO molecules | - | 2 | - | 1 |
| Ligand/ABA molecules | - | - | 1 | - |
| Ligand/GOL molecules |  |  |  |  |
| Water molecules | 72 | 99 | 100 | 77 |
| Average B factor (Protein / Ligand) | 52.0 / - | 37.6 / 39.4 | 38.0 / 38.5 | 46.2 / 48.7 |
| Rmsd Bond lengths (Å) / angles (º) | 0.007 / 0.98 | 0.007 / 0.88 | 0.007 / 0.84 | 0.008 / 0.97 |
| Ramachandran plot statistics | 97.4 % in favoured 0.0 % outliers | 98.9 % in favoured 0.0 % outliers | 97.9 % in favoured 0.0 % outliers | 97.4 % in favoured 1.1 % outliers |
